# Age, Cognitive Factors, and Acceptance of Living with the Disease in Rheumatoid Arthritis: The Short-Term Perspective

**DOI:** 10.3390/ijerph19053136

**Published:** 2022-03-07

**Authors:** Daniel Pankowski, Kinga Wytrychiewicz-Pankowska, Ewa Pisula, Andrzej Fal, Bartłomiej Kisiel, Ewa Kamińska, Witold Tłustochowicz

**Affiliations:** 1 Department of Health and Rehabilitation Psychology, Faculty of Psychology, University of Warsaw, 00-927 Warsaw, Poland; kwytrychiewicz@gmail.com (K.W.-P.); ewa.pisula@psych.uw.edu.pl (E.P.); 2 Institute of Psychology, University of Economics and Human Sciences in Warsaw, 01-043 Warsaw, Poland; 3 Department of Public Health, Medical University of Wrocław, 51-618 Wrocław, Poland; amfal@wp.pl; 4 Warsaw Faculty of Medicine, Collegium Medicum, Cardinal Stefan Wyszyński University, 01-938 Warsaw, Poland; 5 Clinical Research Support Center, Military Institute of Medicine in Warsaw, 04-141 Warsaw, Poland; bkisiel@wim.mil.pl; 6 Department of Rheumatology, Provincial Integrated Hospital in Płock, 09-400 Płock, Poland; awezuzia@mp.pl; 7 Department of Internal Diseases and Rheumatology, Military Institute of Medicine, 04-141 Warsaw, Poland; wtlustochowicz@wim.mil.pl

**Keywords:** acceptance, rheumatoid arthritis, vascular diseases, diabetes, longitudinal study

## Abstract

Rheumatoid arthritis is a chronic inflammatory disease leading to disability, reduced quality of life, and severe depressive symptoms. Theoretical models and research emphasize the importance of cognitive factors such as illness-related beliefs and cognitive appraisals in the process of adapting to life with a chronic disease. Objectives: The aim of this study was to analyze the role of age, disease duration, and cognitive factors in the level of acceptance of life with rheumatoid arthritis and determine the factors responsible for short-term (one week) changes without the use of interventions. We also assessed differences in predictors between rheumatoid arthritis, vascular diseases, and diabetes. Methods: Data were collected using a panel study. The first part of the analysis included 83 participants who declared a medical diagnosis of rheumatoid arthritis. In the second part of the analysis, in addition to people with rheumatoid arthritis (69 participants), two control groups were also included: diabetes (n = 26) and vascular disease (n = 26). The analysis examined basic sociodemographic and clinical data, cognitive appraisals, illness-related beliefs, and acceptance of living with the disease twice in one week. Results: The relationship between age and levels of acceptance of living with the disease was cubic, but the groups distinguished based on age and disease duration did not differ in terms of the analyzed variables. Cognitive appraisals (both baseline and changes over one week) were responsible for changes in acceptance of living with the disease, although other variables (sociodemographic, clinical, and illness-related beliefs) also played a role. The predictors of change in acceptance of living with the disease differed between analyzed diagnoses. Conclusions: Cognitive factors are an important aspect of the adaptation process to living with an illness. Potential clinical applications and future directions of research are discussed.

## 1. Introduction

The growing number of people struggling with chronic diseases is a serious global issue [1]. Epidemiological data show that these types of problems affect not only the elderly, but also young people, resulting in, for example, negative impacts on their ability to perform paid work, fulfill social roles, and quality of life [2]. These types of difficulties may additionally increase the risk of other disorders, particularly mental health issues such as depression [3,4]. For this reason, it is extremely important to study factors, both modifiable (such as psychological factors) and non-modifiable (such as age), that can help chronically ill people adapt and reduce their burden.

Adapting to life with a chronic disease is a long and complicated process. The extent to which a person adjusts to living with a disease is most often described by positive (e.g., acceptance of living with the disease; ALD) and negative (severity of depressive or anxiety symptoms) indicators [5], and these indicators are of great importance for a patient’s quality of life [6,7] and adherence to medical regimens [8]. It is worth emphasizing that a high degree of adaptation is not constituted by a lack of depressive or anxiety symptoms, but should also be characterized by high levels of positive indicators, such as ALD.

Levels of ALD have been analyzed in many groups of patients. In patients with chronic heart failure, it has been observed that reduced health-related quality of life may contribute to difficulties accepting the disease, which, in turn, result in diminished involvement of the patient in the treatment process [9]. Similar conclusions were drawn by Bień et al. [10] in the context of pregnant diabetes patients: their results indicated that higher ALD contributes to a better quality of life and perception of health. Research conducted with elderly people indicated that patients’ depression symptoms and functional and cognitive status are correlates of ALD [11]. In turn, in a study conducted on a group of diabetic patients, Bąk et al. [12] noticed that sexual dysfunction is related to level of acceptance of the disease. On the other hand, Janowski et al. [13] noted that in patients with lower back pain, the appraisals of the disease, such as obstacle, threat, or illness importance, play a very important role in ALD. However, to date, few studies have investigated factors related to ALD levels in people with RA and its cognitive correlates [14].

Rheumatoid arthritis (RA) is a chronic inflammatory disease that affects approximately 1% of the population and is much more common in women than in men. The most common symptoms of RA are pain, morning stiffness, and swollen joints [15]. The course of RA varies over time and between individuals. Scott and Steer [16] identify three indicators to define the course of RA: the impact of joint inflammation, the effect of RA on general health, and its effect on joint damage. The disease is progressive, with increasing difficulties in daily functioning [17], leading over time to disability. Numerous studies indicate, inter alia, a reduced level of quality of life [18] and a high intensity of depression and anxiety symptoms [19]. In addition to clinical factors that may contribute to the deterioration of the indicators of adaptation to the disease [20], psychological variables also play an important role, including beliefs related to the disease [21], self-efficacy, and social support [22]. However, as noted earlier, little attention has been paid to the study of ALD in this group of patients. Kostove et al. [23], on the basis of interviews conducted with a group of 20 people with RA, distinguished five periods of acceptance of living with the disease (naming the illness, realizing the illness, resisting the illness, ‘hitting the bottom’, and integrating the illness), while pointing out that acceptance of living with illness is not a linear process in this group of patients. In turn, Paloș and Vîscu [14] observed that unconditional self-acceptance is negatively correlated with both psychological and somatic anxiety as well as with automatic negative thoughts. In further research, it is worth considering factors that would be susceptible to modification by various types of interventions.

The coping with disease model of Maes, Leventhal, and de Ridder [24] and the common-sense model of illness (CSM; Leventhal et al. [25]) are frequently used to analyze this process of adaptation. Both of these approaches attribute significant roles to cognitive factors: cognitive appraisals (CAs) and cognitive representations of the disease/illness-related beliefs (IRBs), respectively. According to Lazarus and Folkman’s [26] stress-coping theory, a person’s relationship to their environment is subject to CA, which addresses elements of the situation that are relevant to the individual’s well-being. In the context of chronic illness, CAs are of critical importance in determining both the emotions experienced and the choice of strategies used to cope with illness-related stress [24]. On the other hand, the CSM of illness elaborates on what illness representations consist of, while providing a conceptual framework that explores the perceptual, behavioral, and cognitive processes that influence health behavior and coping outcomes [27]. In this theoretical framework, IRBs are postulated to cover five key components of a disease: (1) an identity component; (2) a causal component; (3) a time component; (4) a consequence component; and (5) a disease treatment/controllability component [25,28]. The most important difference between these constructs is that CAs are cognitive processes while IRBs provide a cognitive structure.

Previous research has shown that IRBs can change over time and are related to mental health [29], while CAs are associated with the effects of the adaptation process (see: [26]) and clinical factors [24]. As mentioned above, adaptation to a chronic disease is a dynamic process, therefore both its indicators and individual components change over time. In addition to non-modifiable variables such as age or gender, theoretical models distinguish a number of factors that may be the subject of therapeutic work. The vast majority of studies focus on assessing the effectiveness of selected interventions in the short term (immediately after it ends) and long term (e.g., 6 months later; [30]). However, little is known about how the adaptation process proceeds; in particular, about the changes that may occur in IRBs or CAs attributed to the disease in the short term, without the use of therapeutic interventions. There is still not enough data in the literature on this subject. This gap could be filled by an in-depth analysis of so-called “waiting list” groups, but in the literature they are only treated as a reference point for the experimental and placebo groups. Similarly, also in a short-term perspective, statistically significant changes in the scope of constructs, which are often described as stable over time, cannot be expected, which may additionally contribute to the failure of previous studies to address this issue. IRBs may change over time [31] and depend on the information received by patients (e.g., via social media or contact with other patients) and whether they can be modified; thus, the formation of beliefs is dynamic. Cognitive appraisals of diseases may also change over time, being sensitive to changes in the patient’s clinical condition, coping strategies used, assessment of one’s resources, previous adaptation effects, etc. [24]. Due to this fact, it should be assumed that cognitive factors can influence the level of adaptation over the course of days or weeks, independent of any intervention. Such changes may suggest directions for interventions for patients, particularly informational/educational interventions. This is particularly important in countries or regions where access to psychological care is difficult for people suffering from chronic diseases.

Taking into account all the above information, the study adopted four research objectives:

- Research objective 1: Describe the relationship between age, duration of the disease, and levels of ALD in RA patients.
- Research objective 2: Describe the relationships between modifiable cognitive variables and levels of ALD.
- Research objective 3: Assess the stability of IRBs and CAs in the short term.
- Research objective 4: Identify ALD predictors in RA in the short-term perspective and compare them between different medical diagnoses.

## 2. Method

### 2.1. Participants and Procedure 

Due to the pandemic and the resulting difficulties faced by health services, the data were collected using a panel study and repeated measurements were taken between the 23 February 2021 (T1) and 03 March 2021 (T2). Inclusion criteria were: a declared medical diagnosis of rheumatoid arthritis (RA), vascular diseases (VD), or diabetes (D); being aged above 18 years; declaring the absence of mental disorders (e.g., depression); and having no oncological diseases. In the case of comorbidities, participants were asked to take into account only the above-mentioned diseases in their responses. The purpose of the study was blinded and all participants gave informed consent. The study was approved by the Local Ethics Board.

The first measurement was performed on a sample of 83 participants declaring a medical diagnosis of RA; it included 35 men (42.2%) and 48 women (57.8%). The mean age of the respondents was 59.66 (SD = 13.58) and the mean duration of illness was 11.20 (SD = 8.89). 

Repeated measurement was performed on a total of 121 people: 69 RA participants and 2 control groups—vascular diseases (VD; n = 26) and diabetes (D; n = 26). The choice of these diseases was based on their high prevalence in the general population, diverse age at disease onset, as well as a similar gender prevalence. The study sample was 41.3% women (RA: 53.6%; VD: 34.6%; D: 15.4%) and the mean age was 60.66 (SD = 14.6) years—RA: 58.91 (SD = 13.75); VD: 62.65 (SD = 13.88); D: 63.31 (SD = 15.34). The mean duration of the disease in the analyzed sample was 12.4 (SD = 9.84) years—RA: 11.07 (SD = 8.19); VD: 17.42 (SD = 13.68); D: 10.88 (SD = 7.81). The mean number of hospitalizations during the last 12 months was 0.34 (SD = 0.80)—RA: 0.054 (SD = 0.98); VD: 0.12 (SD = 0.43); D: 0.04 (SD = 0.20). 

### 2.2. Questionnaires

#### Sociodemographic Variables: Gender (Male/Female/Other); Age in Years

Clinical variables: Time since diagnosis of the disease (in years); number of hospitalizations in the last 12 months.

The Illness-Related Beliefs Questionnaire (IRBQ; [32]; Supplementary File S1) was used to assess the intensity of the patient’s personal beliefs about key aspects of their chronic disease. Thirteen IRBs were assessed on a continuum ranging from 1 to 10. 

Illness-Related Appraisals Scale-Revised [33]; Supplementary File S2): this self-report scale assesses the following appraisals: loss (T1 α = 0.92; T2 α = 0.93), harm (T1 α = 0.93; T2 α = 0.94), benefit (T1 α = 0.85; T2 α = 0.92), challenge (T1 α = 0.89; T2 α = 0.92), value (T1 α = 0.87; T2 α = 0.90), threat (T1 α = 0.90; T2 α = 0.95), and importance (T1 α = 0.86; T2 α = 0.84).

The Acceptance of Life with the Disease Scale (ALDS; [34]) was used to measure the degree of acceptance of one’s life with a disease. It consists of three subscales: satisfaction with life despite the disease (T1 α = 0.87; T2 α = 0.87); reconcilement with the disease (T1 α = 0.87; T2 α = 0.88); self-distancing from the disease (T1 α = 0.84; T2 α = 0.90); and global score (T1 α = 0.93; T2 α = 0.93).

### 2.3. Statistical Analysis

First, outliers were assessed in terms of age using box plots. The next step was to determine the relationship between age and ALD using curve estimation in RA patients. 

In the next step, the confirmatory factor analysis (CFA) of the scales used in the cross-sectional study was performed. This allowed the derivation of the statistically strongest weighting combination of the individual variables in each category to form a latent variable in structural equation modeling (SEM). Confirmatory factor analytic models were applied and tested in a stepwise manner for each of the five latent variables separately. First, individual parameters within each of the construct models (e.g., factor loadings) were evaluated for significance at the *p* < 0.05 level. Minor adjustments were applied to the models to arrive at a final factor structure for each of the analyzed constructs.

Subsequently, SEM was carried out using the latent variables identified in the first step. The overall objective of the modeling was to develop a relatively parsimonious representation of the information that would maximize the model fit while judiciously utilizing available degrees of freedom. The IBM AMOS 27 program was used in the calculations.

The next step was to assess the significance of differences between T1 and T2 in terms of IRBs, CA, and ALD both in the whole group and in the subgroups distinguished by diagnosis. For this purpose, the *t*-test for dependent samples was used. Further, the interactions between predictors of ALD were calculated using the “jtools” R package [35]. The next step was to determine the predictors of changes in ALD. In order to do so, stepwise regression analyses were performed in which the dependent variables were T2-T1 differences in ALDS global score and subscales. Sociodemographic and clinical variables, CAs (T1), IRBs (T1), and changes in CAs (T2-T1) and IRBs (T2-T1) were analyzed as factors that may be responsible for changes in ALD. Reliability was assessed using Cronbach’s alpha. The analyses were performed with SPSS 27.0.1.0 and RStudio.

## 3. Results

**Figure 1:**
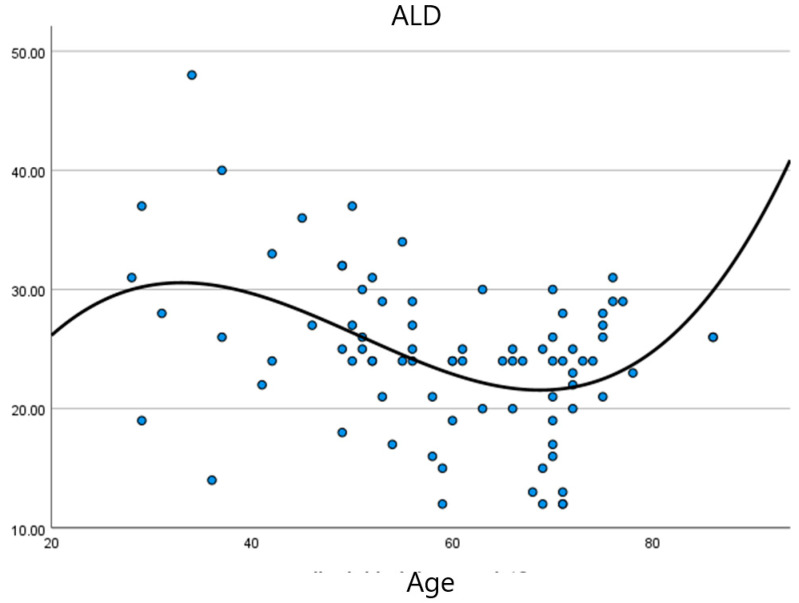
Relationship between ALD and age. ALD—Acceptance of living with the disease.

First, the relationship between age and ALD was explored using curve estimation (Figure 1). 

To analyze the relationship between ALD and age, curve estimation was used, which showed that the most accurate model was the cubic model (*R*^2^ = 0.20; F (3.79) = 6.72; *p* < 0.001). Further analyses showed no relationship between the duration of illness and the level of ALD, and again the cubic model turned out to be the best fitted model (*R*^2^ = 0.03; F (3.79) = 0.77; *p* > 0.05).

The models used to separately develop the constructs representing IRBs (subscales: disease, control, and social), CAs, and ALD all resulted in a satisfactory fit to the observed correlations (see Table 1).

Table 2 shows the standardized factor loadings for each of the analyzed variables (see also Supplementary Files S3–S8).

Three IRB factors were distinguished: IRB-Disease, IRB-Control, and IRB-Social. In the case of IRB-Control, despite the good model fit parameters, the factor loading remained statistically insignificant, so this latent variable was not included in the analyses. On the other hand, in the case of CA, two assessments—value and benefit—were removed from the model to improve its fit. In the case of ALD, three factors were used in accordance with the original assumptions of the scale, which were then placed in the models as explained variables.

**Figure 2:**
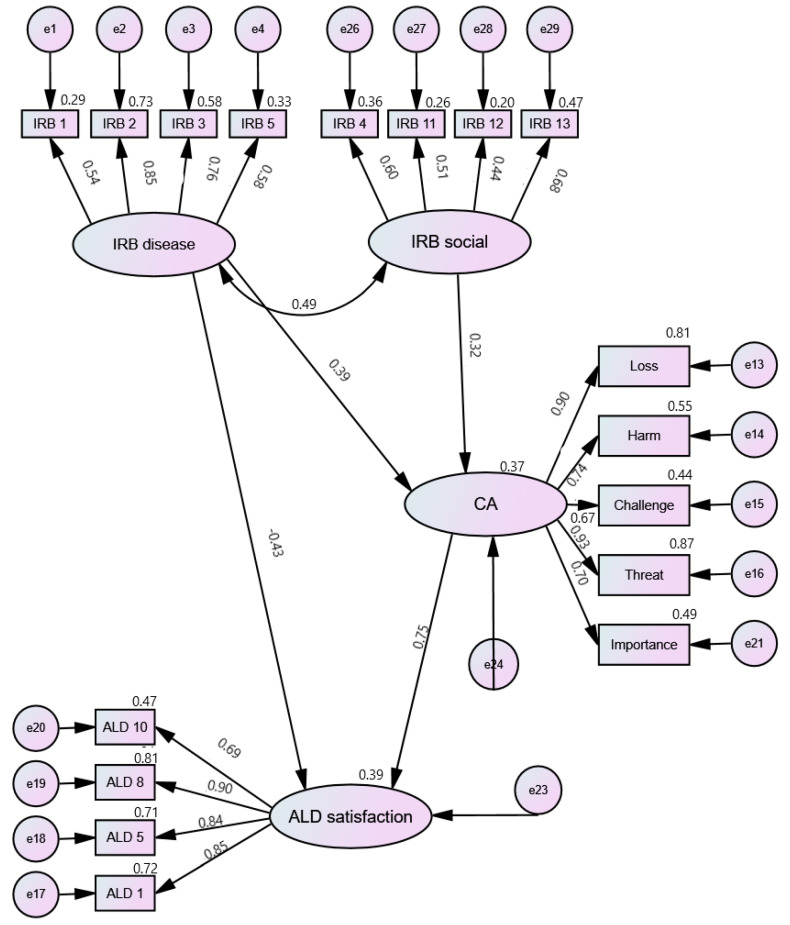
Cognitive factors and ALD Satisfaction: Path diagram. IRB—Illness-related Beliefs; CA—Cognitive Appraisal; ALD—Acceptance of Living with the Disease.

**Figure 3:**
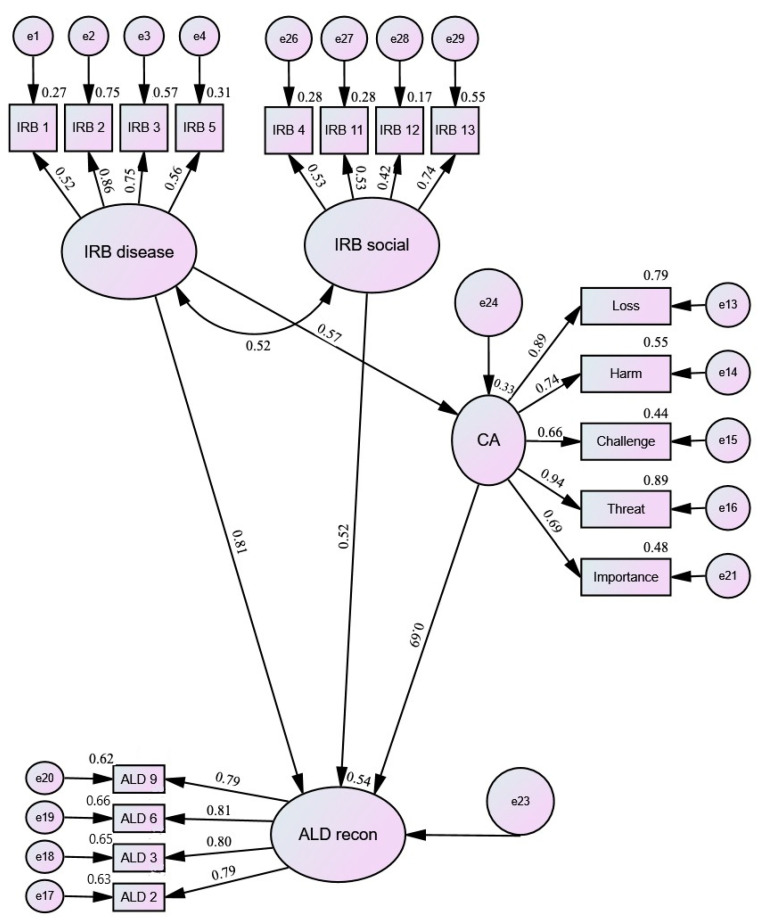
Cognitive factors and ALD Reconcilement: Path diagram. IRB—Illness-related Beliefs; CA—Cognitive Appraisal; ALD recon—Acceptance of Living with the Disease: Reconcilement.

**Figure 4:**
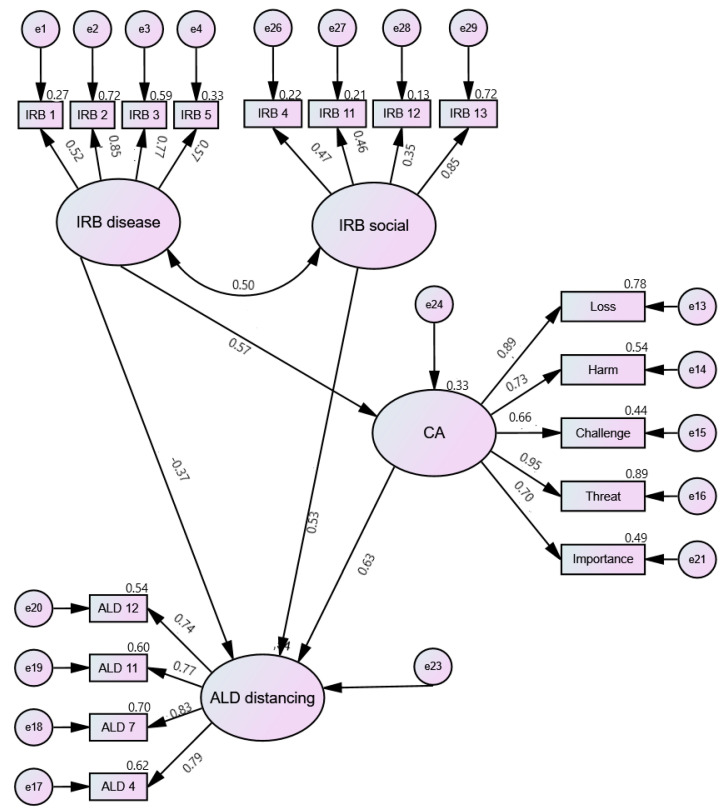
Cognitive factors and ALD Distancing: Path diagram. IRB—Illness-related Beliefs; CA—Cognitive Appraisal; ALD—Acceptance of Living with the Disease.

For each of the models, the solutions were assessed and a number of solutions were tested, first using the significance level of regression weights and then the model fit parameters. A number of models were tested to assess both the direct and indirect (via CA) influence of IRBs (Disease and Social) on levels of ALD. The best fit models are shown below (Figure 2, Figure 3 and Figure 4). The model fit parameters are presented in Table 3.

Reconcilement and Distancing had the same path pattern: IRB-Disease had both direct and indirect (via CA) effects on these ALD components; IRB-Social and CA had direct effects on ALD. For ALD-Satisfaction, the best model was that in which IRB-Disease had both direct and indirect (via CA) effects on ALD; IRB-Social had an indirect effect (via CA) on ALD.

T2-T1 correlations between the analyzed variables are presented in Supplementary File S9. The analysis of the repeated measurement data began with a comparison of the mean IRBs, CAs, and ALDs between measurements 1 and 2 (Table 4).

The analyses showed that there were statistically significant differences between T1 and T2 in terms of IRB1 (relating to the duration of the disease), IRB12 (relating to embarrassment), and benefit (CA). On the other hand, the analyses carried out in the subgroups distinguished according to diagnosis showed that for people with diabetes, a statistically significant difference in IRB 1 (reduction at T2) and ALD-Satisfaction (increase at T2) was observed. In people with RA, statistically significant differences were found in IRB 4 (relating to the visibility of symptoms; decrease at T2) and IRB 12 (increase at T2). There were no statistically significant differences in the VD group (see Supplementary File S10 for details). However, it should also be noted that the analyzed groups were small, so the differences might not be statistically significant.

In the next step, interactions between consecutive ALDS predictors (global score and subscales) and medical conditions were analyzed. The results (see Supplementary Files S11–S15) showed statistically significant interactions between IRBs regarding one’s condition improving (T1 and T2-T1), visibility of symptoms (T1), the possibility of predicting the course of the disease (T1), the possibility of obtaining help from medical personnel (T1), how embarrassing the disease is (T1), the effectiveness of treatment (T2-T1), the attitude of other people to people with this disease (T2-T1), the belief regarding the comparison of disease severity with other people (T2-T1), as well as the following CAs: harm (T1 and T2-T1), importance (T1 and T2-T1), challenge (T2-T1), threat (T2-T1), age, and hospitalizations. 

In the next step, stepwise regression was computed with changes in ALDS global score and subscales as dependent variables. As independent variables, sociodemographic and clinical variables (T1) as well as cognitive variables (CAs and IRBs)—both baseline values (T1) and differences between T2 and T1—were introduced into the models (Table 5). 

The percentage of variance explained ranged from 8.9% (ALD-Reconcilement; all groups) to 58.9% (ALD-Satisfaction; VD group). Depending on the analyzed group, sociodemographic and clinical variables, IRBs, CAs, and changes therein were statistically significant predictors of changes in ALD. The analyses showed that changes in CAs and IRBs explained the changes in levels of ALD to a greater extent than their baseline values (T1). Change in the Importance CA was a positive predictor of: change in ALD global score in the whole group analyzed together and the diabetes group; change in ALD-Satisfaction in the whole group; change in ALD-Reconcilement in diabetes; and change in ALD-Satisfaction in the whole group and the RA group. Threat was a positive predictor of change in ALD global score in the VD group. Change in threat was a positive predictor of change in ALD global score in RA patients. Change in threat was also a positive predictor of change in ALD-Satisfaction and ALD-Reconcilement in RA, and in ALD-Distancing in RA patients.

## 4. Discussion

The study focused on factors related to levels of acceptance of living with the disease (ALD) in people with rheumatoid arthritis (RA). Modifiable cognitive factors—cognitive appraisals (CAs) and illness-related beliefs (IRBs)—as well as sociodemographic variables (sex, age), and clinical factors (the duration of the disease) were assessed. We found a cubic relationship between age and ALD, and the paths between cognitive factors and levels of acceptance differed depending on the ALD subscales. Further analyses carried out on data collected after one week showed that there were statistically significant differences between the measurements in IRBs, Cas, and ALD, and that sociodemographic, clinical, and cognitive variables were responsible for changes in ALD in people who had not been treated with therapeutic interventions. We also observed statistically significant interactions between the diagnoses’ predictors, which may have practical implications.

The first part of the analyses focused on determining the relationship between age and ALD. The fitted curve indicated a cubic relationship. The highest levels of ALD were observed in younger people, while levels decreased with age, flattening out and rising around the age of 70. One explanation for this observation may be that, with age, the disease may have a negative impact on social functioning, influencing spheres of life such as work, family, and the pursuit of interests [2,36]. On one hand, people retire after 60–65 years of age, and on the other hand, they live with the disease for a longer time and might become better adapted to it. It should be noted that RA has a peak incidence around the age of 50 [15]. 

The next part of the analysis focused on the identification of interrelationships between modifiable cognitive factors related to levels of acceptance of living with rheumatoid arthritis. First, latent variables were determined with the use of confirmatory factor analysis, and then models were tested in which the relationships between these variables were fitted. To obtain the best fit, it was necessary to remove the Value and Benefit scales for CA. It is worth noting that the tested models confirmed the two-factor structure of the scale (positive CAs, i.e., value, benefit, and challenge, vs. negative ones), which had satisfactory fit parameters. However, due to the nature of the analyses and better fit parameters, a one-factor model was used for structural equation modeling (SEM), which did not contain the above two scales. Additionally, despite good fit parameters, the regression weights remained insignificant for IRB-Control, which may be due to the small sample size [37]. The next step was to test a number of models: in the case of ALD-Reconcilement and ALD-Distancing, identical paths between the analyzed variables were found; IRB-Disease had both direct and indirect (via CA) effects while IRB-Social and CA had only a direct effect on acceptance. On the other hand, for ALD-Satisfaction, the best fitted model turned out to be the one in which IRB-Disease had both direct and indirect (via CA) effects and IRB-Social had an indirect impact (via CA). It is also worth paying attention to the model fit coefficients, which had average fit. It should be noted here that the sample on which the analyses were performed was relatively small (83 observations). Hu and Bentler [38] found that in small samples it is possible that the model fit parameters might overreject the true model.

These results are important: they show that ALD is a multidimensional construct, the individual components of which are shaped on the basis of other dependencies between variables; it is not one-dimensional, as is apparent from factor analyses carried out on the acceptance of illness scale (AIS; [39]). These results can also be used in clinical practice. Based on the theoretical models of Maes et al. [24] and Leventhal et al. [25,27], it is possible to determine the relationships between IRBs and CAs, which, in the light of this research, play a very important role in the process of adaptation to life with a chronic disease. These analyses may also indicate directions for potential therapeutic interactions within, for example, cognitive behavioral therapy, which may help increase acceptance of living with RA. 

The analyses also show statistically significant differences in terms of both IRBs and CA or ALD between two measurements performed one week apart. These differences can be observed both in the entire sample and in subgroups distinguished based on diagnosis. It is worth noting that the numbers for the subgroups were small, and the size of the effect may suggest that with increasing samples, the significance level could be *p* < 0.05. Cognitive factors, due to their dynamic nature and sensitivity to changes occurring both in connection with the disease itself (such as exacerbation of symptoms) and environmental factors (new information, social support, etc.), may lead to changes in the indicators of adaptation to the disease. It should be emphasized that interventions are not necessary for such changes to occur—they may occur over time under the influence of many, often random, factors.

Our analyses suggest that predictors of short-term change in ALD differ across diagnoses, as well as in the cognitive factors that play an important role in this process. Difference in importance (T2-T1) as well as threat (T1 and T2-T1) were positive predictors of change in ALD for most diagnoses. Benefit contributed to lower acceptance in the case of ALDS global score and self-distancing from the disease (all diagnoses), and had a beneficial effect on satisfaction with life despite the disease and self-distancing from the disease (RA). In summary, the appraisal of the disease, even when negative, can contribute to greater acceptance. On the other hand, beliefs regarding control over the onset of the illness and its course were linked to lower levels of ALD. Beliefs concerning, inter alia, the level of one’s own knowledge about the disease or its chronic nature may translate into greater acceptance. 

Interactions (predictors × diagnoses) clearly showed that there were large differences between the specific diagnoses. Of particular importance to therapeutic work (e.g., cognitive behavioral therapy), is the observation that both IRBs and cognitive appraisals may have different effects on ALD depending on the diagnosis. One example is belief regarding one’s condition improving (IRB 3), which is beneficial for both people with RA and D, but has a negative impact in the short term on the level of acceptance in people with VD. These results can be taken as an argument for greater personalization of therapy based on diagnosis and further exploration of the role of both beliefs and cognitive appraisals. 

Developing knowledge about change in a short-term perspective is also particularly important: some processes, such as cognitive appraisals, are dynamic and their intensities may change over a short period of time depending on, for example, the patient’s mood or events in their lives. Another argument for analyzing these processes in the short term, especially in the “natural environment”, is the fact that research involving interventions aimed at modification of beliefs may affect the degree of adaptation to living with the disease not only by changing certain IRBs, but also through the therapist effect, and the changes may be related to new knowledge or experiences of other patients, as well as the therapeutic groups that provide social support. On the other hand, the results of research conducted with inpatients may be distorted due to conditions in wards (lack of privacy, or problems with sleep or adaptation to a new place), expectations regarding new treatments, separation from one’s family, etc.

The results of this study also suggest that the cognitive representation of a disease is more complex and consists of more than five key beliefs [28], especially when comparing medical diagnoses with each other. The results indicate that IRBs regarding knowledge or perception of the disease by other people are statistically significant predictors of changes in ALD. In further research, it is also worth identifying and analyzing the role of diagnosis-specific IRBs due to their unique nature (for details see [40]).

For therapeutic interventions, the SEM results may be of the greatest importance, indicating that the cognitive appraisals of a disease can be modified by changing beliefs, and that both of these factors play a large role in ALD. This is especially important due to the fact that adaptation to living with the disease is constituted not only by the absence of depressive or anxiety symptoms, but also by high levels of positive indicators (see also: [41]). It is also worth emphasizing that different diagnoses have different predictors for ALD. Therefore, it is important to match specific therapeutic strategies to a given diagnosis, as this can improve short-term as well as long-term outcomes. The data can also be used in information campaigns in the form of posters/brochures or infographics distributed via social media. Such materials could better shape the beliefs of sick people, indirectly increasing the sense of acceptance. As mentioned earlier, IRBs are dynamic and may depend on many factors related to both information coming from one’s environment—both from authorities (e.g., doctors) and less reliable sources (e.g., internet forums)—but also may be related to the severity of the symptoms of the disease, the effectiveness of the treatment, and any side effects. Therefore, it is very important to support patients in developing specific ways of thinking about their own disease and the factors related to it. This is especially important for RA patients. For these people, the disease is progressive, with pain as the predominant symptom. With time, people with this diagnosis may have problems with moving around and performing daily tasks, their quality of life deteriorates, and depressive symptoms worsen; therefore, it seems necessary to foster beliefs that may increase levels of ALD. 

## 5. Limitations and Further Directions

This study also has some limitations. It was conducted online, so it was not possible to medically confirm the diagnoses; however, other studies indicate a very high rate of agreement between declared and actual diagnoses (as high as 99%; [42]). Furthermore, other clinical factors, such as the severity of the disease, were not sufficiently controlled (e.g., using DAS-28 for RA patients; [43]). Future research should consider a wider range of variables, such as, inter alia, health burdens [44,45], cognitive impairment, or economic opportunities (see also: [46]), but in the current research this was not possible due to the study procedure as well as constraints imposed by the pandemic. The results show a slight convergence between predictors in the RA group and in the overall group, which may be due to the larger size of the RA group. The study also did not analyze people with type 1 and type 2 diabetes separately. Subsequent studies could consider both a broader repertoire of diagnoses (e.g., skin or respiratory diseases) as well as objective health status indicators (such as C-reactive protein levels in RA patients). 

Another issue is the psychometric properties of the tools used: the cognitive constructs controlled in the study may change over time and depend on many factors [24]; see also Supplementary File S9 for T1 and T2-T1 correlation matrix between measured variables), some of which were beyond our control. We do not know whether the respondents experienced any deterioration in their health or what sources of information they used during the week (which is especially important for IRBs; [47]. This topic is also the subject of another publication describing the psychometric properties of the tools (in preparation). A proper solution to this problem would be a diary study in which these types of variables could be controlled; this would allow us to better identify both the dynamics of changes and possible factors that may affect them. Both the obtained results and the potential expansion of knowledge in the field of belief stability, cognitive appraisals, and factors responsible for change therein over time without the use of an intervention can also be widely used in the assessment of therapy stability (changes in cognitive factors or adaptation indicators after the end of the intervention).

## 6. Conclusions

Relationship: Our results showed a cubic relationship between age and ALD. We found that the pathways between cognitive factors and ALD differed depending on the ALD subscale. Levels of certain IRBs and CAs as well as ALD varied over the course of a week without the use of therapeutic interventions; sociodemographic and clinical factors and, primarily, IRBs and CAs were responsible for these changes in ALD. The three diagnoses examined differed in terms of the statistically significant predictors of changes in ALD. This highlights the importance of targeted interventions and suggests possible directions for them.

## Figures and Tables

**Table 1:** Confirmatory factor analytic results for the development of latent variables.

Latent Variable	χ^2^	*p*	GFI	CFI	TLI	RMSEA
IRB-Disease	0.86	>0.05	0.995	1000	1.038	0.000
IRB-Social	4.587	>0.05	0.975	0.934	0.803	0.126
IRB-Control	3.939	>0.05	0.982	1000	1.426	0.000
CA	1.102	>0.05	0.995	1000	1.032	0.000
ALD-Reconcilement	3.228	>0.05	0.982	0.993	0.979	0.087
ALD-Satisfaction	0.502	>0.05	0.997	1000	1.024	0.000
ALD-Self-distancing	1.741	>0.05	0.989	1000	1.005	0.000

**Table 2:** Standardized factor loadings for all latent variables.

	IRB-Disease	IRB-Control	IRB-Social	CA	ALD-Satisfaction	ALD-Reconcilement	ALD-Distancing
IRB 1	0.58						
IRB 2	0.77						
IRB 3	0.83						
IRB 5	0.58						
IRB 6		−0.02					
IRB 7		0.26					
IRB 8		0.25					
IRB 9		0.6					
IRB 10		0.44					
IRB 4			0.55				
IRB 11			0.56				
IRB 12			0.53				
IRB 13			0.64				
CA: Loss				0.91			
CA: Harm				0.75			
CA: Challenge				0.67			
CA: Threat				0.92			
CA: Importance				0.68			
ALD 1					0.86		
ALD 5					0.85		
ALD 8					0.89		
ALD 10					0.68		
ALD 2						0.81	
ALD 3						0.82	
ALD 6						0.84	
ALD 9						0.78	
ALD 4							0.78
ALD 7							0.85
ALD 11							0.78
ALD 12							0.75

**Table 3:** Summary of the models and associated goodness-of-fit indices.

ALD Subscales	χ^2^	df	*p*	Normed χ^2^ Value	CFI	TLI	AIC	BIC
ALD-Reconcilement	198.769	114	<0.001	1.744	0.877	0.854	276.769	371.103
ALD-Satisfaction	186.682	114	<0.001	1.638	0.896	0.876	264.682	359.017
ALD-Distancing	177.89	114	<0.001	1.56	0.903	0.884	255.89	350.225

**Table 4:** Differences between T1 and T2.

Variables	T1		T2		*t*	*p*	Cohen’s *d*
	M	SD	M	SD			
IRB 1	8.76	1.65	8.32	2.03	2.137	0.035	0.194
IRB 2	7.83	1.97	7.54	2.15	1.424	0.157	0.129
IRB 3	7.36	2.06	7.06	2.18	1.700	0.092	0.155
IRB 4	4.76	2.70	4.58	2.81	0.800	0.425	0.073
IRB 5	6.40	2.42	6.39	2.47	0.079	0.937	0.007
IRB 6	7.83	1.75	7.93	1.81	−0.696	0.488	−0.063
IRB 7	4.34	2.86	4.69	2.76	−1.772	0.079	−0.161
IRB 8	5.07	2.69	5.06	2.74	0.061	0.952	0.006
IRB 9	5.34	2.66	5.12	2.52	0.880	0.380	0.080
IRB 10	4.88	2.29	5.07	2.30	−0.745	0.458	−0.068
IRB 11	3.82	2.28	4.09	2.44	−1.328	0.187	−0.121
IRB 12	2.69	2.43	3.12	2.77	−2.298	0.023	−0.209
IRB 13	4.31	2.47	4.53	2.38	−1.036	0.302	−0.094
Loss	14.31	5.09	13.95	5.25	1.060	0.291	0.096
Harm	12.06	5.09	12.03	5.43	0.077	0.938	0.007
Benefit	7.79	3.15	8.55	4.16	−2.577	0.011	−0.234
Challenge	16.01	4.57	15.55	4.71	1.433	0.154	0.130
Value	13.35	4.51	13.64	4.66	−1.025	0.308	−0.093
Threat	15.56	4.25	14.88	4.93	2.134	0.035	0.194
Importance	16.07	4.27	15.93	4.59	0.381	0.704	0.035
ALD-Satisfaction	7.88	2.25	8.12	2.38	−1.745	0.084	−0.159
ALD-Reconcilement	6.99	1.88	7.06	1.92	−0.487	0.627	−0.044
ALD-Distancing	8.75	2.35	9.04	2.72	−1.434	0.154	−0.130
ALD Global Score	23.63	5.77	24.22	6.20	−1.517	0.132	−0.138

**Table 5:** Predictors of changes in Acceptance of Living with the Disease.

	Statistically Significant Predictors	*R*^2^ for the Model	Adjusted *R*^2^ for the Model	β	*t*	*p*
Acceptance: Global score						
All: (*B* = 0.943; *F* = 7.465; *p* < 0.001)	Importance (T2-T1)	0.161	0.139	0.289	3.359	0.001
	Belief about control over the onset of the disease (T2-T1)			−0.193	−2.251	0.026
	Benefit (T2-T1)			−0.169	−1.982	0.050
RA: (*B* = 0.578; *F* = 10.829; *p* = 0.002)	Threat (T2-T1)	0.139	0.126	0.373	3.291	0.002
VD: (*B* = 3.405; *F* = 8.120; *p* = 0.002)	Belief regarding one’s condition improving (T1)	0.414	0.363	−0.656	−3.824	0.001
	Threat (T1)			0.442	2.580	0.017
D: (*B* = 1.068; *F* = 18.738; *p* < 0.001)	Importance (T2-T1)	0.438	0.415	0.662	4.329	0.000
Satisfaction						
All (*B* = 0.551; *F* = 5.405; *p* < 0.001)	Belief regarding the possibility of predicting the course of the disease (T2-T1)	0.157	0.128	0.152	1.732	0.086
	Belief about control over the onset of the disease (T2-T1)			−0.177	−2.043	0.043
	Female gender			−0.204	−2.355	0.020
	Importance (T2-T1)			0.175	2.004	0.047
RA (*B* = −3.251; *F* = 5.899; *p* < 0.001)	Benefit (T1)	0.214	0.178	0.379	3.290	0.002
	The belief about the duration of the disease (T1)			0.309	2.660	0.010
	Threat (T2-T1)			0.260	2.343	0.022
Vascular (*B* = 0.615; *F* = 18.915; *p* < 0.001)	Belief regarding the possibility of predicting the course of the disease (T2-T1)	0.622	0.589	0.715	5.574	0.000
	Female gender			−0.358	−2.791	0.010
Diabetes (*B* = −0.015; *F* = 9.963; *p* < 0.001)	Belief regarding how embarrassing the disease is (T1)	0.576	0.518	0.535	3.850	0.001
	Number of hospitalizations			−0.438	−3.094	0.005
	Belief about control over the onset of the disease (T2-T1)			−0.407	−2.876	0.009
Reconcilement						
All (*B* = 0.141; *F* = 12.732; *p* < 0.001)	Belief about control over the onset of the disease (T2-T1)	0.097	0.089	−0.311	−3.568	0.001
RA (*B* = 0.126; *F* = 14.543; *p* < 0.001)	Threat (T2-T1)	0.178	0.166	0.422	3.814	0.000
Vascular (*B* = 0.089; *F* = 4.875; *p* = 0.037)	Belief about control over the onset of the disease (T2-T1)	0.169	0.134	−0.411	−2.208	0.037
Diabetes (*B* = −0.193; *F* = 12.408; *p* < 0.001)	Importance (T2-T1)	0.519	0.477	0.658	4.549	0.000
	Duration of the disease			0.324	2.236	0.035
Distancing						
All (*B* = −0.096; *F* = 8.361; *p* < 0.001)	Importance (T2-T1)	0.177	0.155	0.352	4.112	0.000
	Benefit (T2-T1)			−0.224	−2.658	0.009
	Duration of the disease			0.189	2.216	0.029
RA (*B* = −2.247; *F* = 6.101; *p* < 0.001)	Importance (T2-T1)	0.371	0.310	0.308	2.614	0.011
	Number of hospitalizations			−0.276	−2.695	0.009
	Female gender			−0.277	−2.694	0.009
	Belief about knowledge about a disease (T1)			0.272	2.611	0.011
	Benefit (T1)			0.247	2.375	0.021
	Threat (T2-T1)			0.253	2.197	0.032
Vascular (*B* = −1.480; *F* = 6.535; *p* = 0.006)	Belief regarding one’s condition improving (T1)	0.362	0.307	−0.432	−2.595	0.016
	Age			0.417	2.505	0.020
Diabetes (*B* = 0.357; *F* = 16.051; *p* < 0.001)	Importance (T2-T1)	0.583	0.546	0.596	4.236	0.000
	Loss (T2-T1)			0.333	2.367	0.027

RA—Rheumatoid arthritis; VD—Vascular diseases; D—Diabetes; T1—baseline measurement; T2—follow-up measurement.

## Data Availability

The data as well as R code that support the findings of this study will be available after publication at the OSF project page.

## Supplementary Materials

The following are available online at https://www.mdpi.com/article/10.3390/ijerph19053136/s1. Supplementary File S1: Illness-Related Beliefs Scale; Supplementary File S2: Illness-Related Appraisals Scale; Supplementary File S3: CFA: IRB Disease; Supplementary File S4: CFA: IRB Social; Supplementary File S5: CFA: CA; Supplementary File S6: CFA: ALD Distancing; Supplementary File S7: CFA: ALD Reconcilement; Supplementary File S8: CFA: ALD Satisfaction; Supplementary File S9: T1-T2 and T1 correlation matrix; Supplementary File S10: Differences between T1 and T2 in subgroups distinguished by diagnosis; Supplementary File S11: Acceptance Global Score and subscales: interactions between predictors; Supplementary File S12: ALD (T2-T1): interactions between predictors; Supplementary File S13: ALD Reconcilement (T2-T1): interactions between predictors; Supplementary File S14: ALD Satisfaction (T2-T1): interactions between predictors; Supplementary File S15: ALD Self-distancing (T2-T1): interactions between predictors.
