# Supplementary material for: Age, Cognitive Factors, and Acceptance of Living with the Disease in Rheumatoid Arthritis: The Short-Term Perspective
Source: Int J Environ Res Public Health. 2022 Mar 7;19(5):3136. doi: 10.3390/ijerph19053136 (PMC8910175; doi:10.3390/ijerph19053136)
Supplement: Supplementary file 1 [file ijerph-19-03136-s001.zip › Supplementary File S1 IRB.pdf]

# Illness-Related Belief Questionnaire

Version P-13

**<u>Instructions:</u>** On the other side of this sheet, you will find 13 pairs of statements concerning **your** illness. Each pair presents two opposing statements about a certain part of the illness, for example:

The symptoms of my illness are not painful at all.

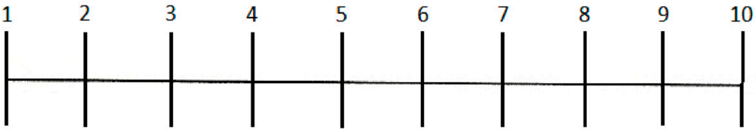


The symptoms of my illness are very painful.

The numbers between these statements indicate how much you agree with the statement on the left or on the right:

closer to 1: you agree more with the statement on the left

closer to 10: you agree more with the statement on the right

**Please read each pair of statements on the other side of the sheet carefully.**

**For each pair, draw a circle around the number that best corresponds to the belief you agree with.**

*Examples:*

The symptoms of my illness are not painful at all.

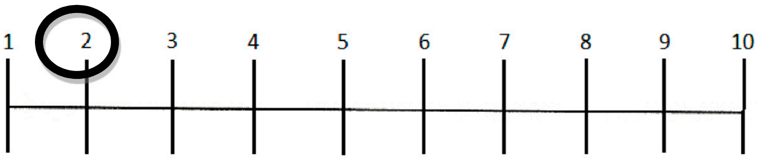


The symptoms of my illness are very painful.

In this example, the answer means that the symptoms of your illness are not very painful.

The symptoms of my illness are not painful at all.

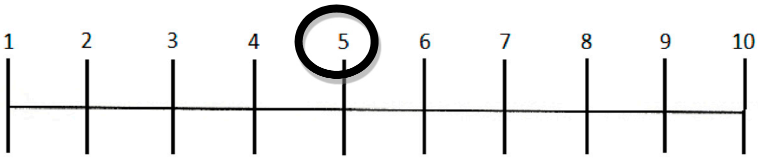


The symptoms of my illness are very painful.

Here, the answer means that the symptoms of your illness are moderately painful.

The symptoms of my illness are not painful at all.

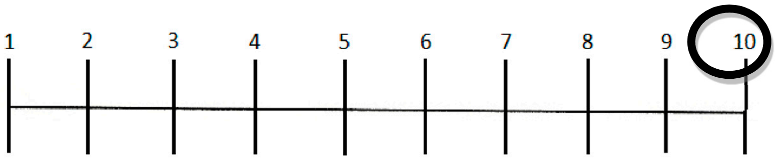


The symptoms of my illness are very painful.

In the last example, the answer means that the symptoms of your illness are very painful.

My illness/health problem: ……………………………………………………

| | 1 | 2 | 3 | 4 | 5 | 6 | 7 | 8 | 9 | 10 | |
|---|---|---|---|---|---|---|---|---|---|---|---|
| My illness will last for a short time. | 1 | 2 | 3 | 4 | 5 | 6 | 7 | 8 | 9 | 10 | My illness will last for a very long time. |
| This illness will not have a significant impact on my life. | 1 | 2 | 3 | 4 | 5 | 6 | 7 | 8 | 9 | 10 | This illness will have a very significant impact on my life. |
| My condition will improve. | 1 | 2 | 3 | 4 | 5 | 6 | 7 | 8 | 9 | 10 | My condition will worsen. |
| The symptoms of my illness are not visible to others. | 1 | 2 | 3 | 4 | 5 | 6 | 7 | 8 | 9 | 10 | The symptoms of my illness are very visible to others. |
| I am able to fully predict the course of my illness. | 1 | 2 | 3 | 4 | 5 | 6 | 7 | 8 | 9 | 10 | I cannot predict the course of my illness at all. |
| I know almost nothing about my illness. | 1 | 2 | 3 | 4 | 5 | 6 | 7 | 8 | 9 | 10 | I know a lot about my illness. |
| I had no control over becoming ill or not. | 1 | 2 | 3 | 4 | 5 | 6 | 7 | 8 | 9 | 10 | Things I have done caused me to become ill. |
| The things I do can influence the course of my illness. | 1 | 2 | 3 | 4 | 5 | 6 | 7 | 8 | 9 | 10 | The things I do have no effect on the course of my illness. |
| The medical staff can influence the course of my illness. | 1 | 2 | 3 | 4 | 5 | 6 | 7 | 8 | 9 | 10 | The medical staff cannot influence the course of my illness. |
| The treatment I get is very effective. | 1 | 2 | 3 | 4 | 5 | 6 | 7 | 8 | 9 | 10 | The treatment I get does not help me at all. |
| In general, others would regard me positively as a person who has this illness. | 1 | 2 | 3 | 4 | 5 | 6 | 7 | 8 | 9 | 10 | In general, others would regard me negatively due to the fact I have this illness. |
| I believe my illness is not embarrassing. | 1 | 2 | 3 | 4 | 5 | 6 | 7 | 8 | 9 | 10 | I believe my illness is embarrassing. |
| Compared to other people who have this illness, my symptoms are very mild. | 1 | 2 | 3 | 4 | 5 | 6 | 7 | 8 | 9 | 10 | Compared to other people who have this illness, my symptoms are very severe. |
