# Supplementary material for: Age, Cognitive Factors, and Acceptance of Living with the Disease in Rheumatoid Arthritis: The Short-Term Perspective
Source: Int J Environ Res Public Health. 2022 Mar 7;19(5):3136. doi: 10.3390/ijerph19053136 (PMC8910175; doi:10.3390/ijerph19053136)
Supplement: Supplementary file 1 [file ijerph-19-03136-s001.zip › Supplementary File S10 Differences between T1 and T2 in subgroups distinguished by diagnosis.pdf]

Supplementary File 10. Differences between T1 and T2 in subgroups distinguished by diagnosis

Table 1. Differences between T1 and T2 in diabetes patients

Variables	T1		T2		t	p	Cohen's d
	M	SD	M	SD			
IRB 1	9.12	1.37	8.08	2.28	2.184	0.039	0.428
IRB 2	8.31	1.41	7.54	1.86	1.882	0.072	0.369
IRB 3	6.69	1.95	6.58	2.08	0.268	0.791	0.053
IRB 4	3.23	2.49	3.62	2.91	-0.664	0.513	-0.130
IRB 5	5.69	2.46	5.96	2.46	-0.484	0.633	-0.095
IRB 6	7.58	1.88	7.46	1.82	0.340	0.736	0.067
IRB 7	5.15	2.80	5.19	2.48	-0.092	0.927	-0.018
IRB 8	3.85	2.63	4.27	2.32	-0.747	0.462	-0.147
IRB 9	4.73	2.39	4.50	2.28	0.690	0.496	0.135
IRB 10	4.77	1.82	4.73	1.76	0.090	0.929	0.018
IRB 11	3.23	2.05	3.69	2.36	-1.364	0.185	-0.267
IRB 12	2.50	2.28	2.85	2.51	-1.434	0.164	-0.281
IRB 13	3.23	1.70	3.88	1.99	-1.739	0.094	-0.341
Loss	14.35	4.55	13.35	4.15	1.653	0.111	0.324
Harm	11.38	5.23	11.77	4.89	-0.693	0.495	-0.136
Benefit	7.27	2.15	8.15	3.26	-1.716	0.098	-0.337
Challenge	15.46	4.35	14.85	4.57	1.039	0.309	0.204
Value	12.73	3.90	12.77	4.24	-0.083	0.934	-0.016
Threat	15.85	3.64	15.35	3.89	0.879	0.388	0.172
Importance	15.35	3.65	15.92	3.73	-0.703	0.489	-0.138
ALD satisfaction	7.88	1.75	8.58	2.14	-2.459	0.021	-0.482
ALD reconcilement	6.85	1.67	7.27	1.76	-1.897	0.069	-0.372
ALD distancing	9.00	2.24	9.31	2.57	-0.829	0.415	-0.163
ALD Global score	23.73	4.89	25.15	5.68	-1.865	0.074	-0.366

Table 2. Differences between T1 and T2 in rheumatoid arthritis patients

Variables	T1		T2		t	p	Cohen's d
	M	SD	M	SD			
IRB 1	8.52	1.74	8.41	1.83	0.462	0.645	0.056
IRB 2	7.78	2.09	7.74	2.13	0.155	0.877	0.019
IRB 3	7.75	1.84	7.43	2.20	1.357	0.179	0.163
IRB 4	5.62	2.47	4.97	2.65	2.581	0.012	0.311
IRB 5	7.00	2.20	6.83	2.24	0.686	0.495	0.083
IRB 6	7.75	1.80	7.91	1.85	-0.770	0.444	-0.093
IRB 7	4.03	2.84	4.36	2.81	-1.402	0.166	-0.169
IRB 8	5.70	2.52	5.16	2.64	1.548	0.126	0.186
IRB 9	5.72	2.57	5.16	2.51	1.709	0.092	0.206
IRB 10	5.35	2.22	5.30	2.33	0.139	0.890	0.017
IRB 11	4.48	2.20	4.41	2.37	0.300	0.765	0.036
IRB 12	2.96	2.49	3.46	2.93	-2.133	0.037	-0.257
IRB 13	4.84	2.51	4.87	2.41	-0.098	0.922	-0.012
Loss	15.23	4.96	14.91	4.93	0.710	0.480	0.085
Harm	12.81	5.00	12.39	5.32	1.001	0.320	0.121
Benefit	8.35	3.66	8.93	4.30	-1.636	0.106	-0.197
Challenge	16.80	4.25	16.30	4.14	1.240	0.219	0.149
Value	14.01	4.63	14.25	4.57	-0.600	0.550	-0.072
Threat	16.01	4.20	15.29	4.78	1.746	0.085	0.210
Importance	17.29	3.96	16.90	4.45	0.801	0.426	0.096
ALD satisfaction	8.30	2.40	8.38	2.42	-0.422	0.675	-0.051
ALD reconcilement	7.29	1.93	7.29	1.93	0.000	1.000	0.000
ALD distancing	9.09	2.37	9.28	2.68	-0.773	0.442	-0.093
ALD Global score	24.68	5.94	24.94	6.20	-0.535	0.594	-0.064

Table 3. Differences between T1 and T2 in patients with vascular diseases

Variables	T1		T2		t	p	Cohen's d
	M	SD	M	SD			
IRB 1	9.04	1.64	8.35	2.33	1.443	0.161	0.283
IRB 2	7.46	2.08	7.00	2.43	1.110	0.278	0.218
IRB 3	7.00	2.51	6.54	2.12	1.251	0.222	0.245
IRB 4	4.00	2.67	4.50	2.97	-0.909	0.372	-0.178
IRB 5	5.54	2.56	5.65	2.90	-0.257	0.799	-0.050
IRB 6	8.27	1.46	8.46	1.61	-0.594	0.558	-0.117
IRB 7	4.35	2.94	5.08	2.86	-1.320	0.199	-0.259
IRB 8	4.65	2.80	5.58	3.26	-1.449	0.160	-0.284
IRB 9	4.92	3.03	5.62	2.71	-0.991	0.331	-0.194
IRB 10	3.77	2.57	4.77	2.66	-1.569	0.129	-0.308
IRB 11	2.65	2.13	3.65	2.65	-1.646	0.112	-0.323
IRB 12	2.19	2.40	2.50	2.55	-0.550	0.587	-0.108
IRB 13	3.96	2.66	4.27	2.55	-0.617	0.543	-0.121
Loss	11.85	5.29	12.00	6.49	-0.174	0.863	-0.034
Harm	10.73	5.02	11.35	6.27	-0.751	0.460	-0.147
Benefit	6.85	2.13	7.92	4.60	-1.267	0.217	-0.248
Challenge	14.46	5.26	14.23	5.93	0.253	0.803	0.050
Value	12.19	4.59	12.88	5.22	-1.000	0.327	-0.196
Threat	14.08	4.75	13.31	6.02	0.895	0.380	0.175
Importance	13.54	4.50	13.35	4.88	0.245	0.808	0.048
ALD satisfaction	6.77	1.97	7.00	2.21	-0.672	0.508	-0.132
ALD reconcilement	6.35	1.85	6.23	1.90	0.306	0.762	0.060
ALD distancing	7.62	2.10	8.15	2.87	-0.925	0.364	-0.181
ALD Global score	20.73	5.30	21.38	6.09	-0.624	0.538	-0.122
