# Supplementary material for: Age, Cognitive Factors, and Acceptance of Living with the Disease in Rheumatoid Arthritis: The Short-Term Perspective
Source: Int J Environ Res Public Health. 2022 Mar 7;19(5):3136. doi: 10.3390/ijerph19053136 (PMC8910175; doi:10.3390/ijerph19053136)
Supplement: Supplementary file 1 [file ijerph-19-03136-s001.zip › Supplementary File S11.pdf]

Supplementary File 11. Acceptance Global Score and subscales: interactions between predictors

Predictors*	Diagnoses	Acceptance Global Score	Satisfaction with Life Despite the Disease	Reconcilement with the Disease	Self-distancing from the Disease
Belief regarding one's condition improving (IRB 3)	VD vs D	t=2.02; p=0.05	n.s.	t=-2.44; p=0.02	t=-2.51; p=0.01
	RA vs VD	t=-3.31; p<0.001	t=-2.65; p=0.01	t=-2.51; p=0.01	t=-2.77; p=0.01
Belief regarding the visibility of symptoms (IRB 4)	VD vs D	t=-2.01; p=0.05	n.s.	n.s.	t=-2.27; p=0.03
Belief regarding the possibility of predicting the course of the disease (IRB 5)	VD vs D	n.s.	n.s.	n.s.	t=-2.10; p=0.04
Belief regarding the possibility of predicting the course of the disease (IRB 8)	RA vs D	t=-2.03; p=0.05	n.s.	n.s.	t=-2.01; p=0.05
Belief regarding the possibility of obtaining help from medical personnel (IRB 9)	RA vs D	t=-2.18; p=0.03	n.s.	n.s.	n.s.
	VD vs D	t=-2.49; p=0.01	n.s.	t=-2.28; p=0.02	t=-2.05; p=0.04
Belief regarding how embarrassing the disease is (IRB 12)	RA vs D	t=-2.44; p=0.02	t=-2.39; p=0.02	n.s.	n.s.
	VD vs D	t=-2.40; p=0.02	t=-2.97; p<0.01	n.s.	n.s.
Belief regarding one's condition improving (IRB 3; T2 - T1)	VD vs D	n.s.	n.s.	t=2.19; p=0.03	n.s.
Belief regarding the possibility of predicting the course of the disease (IRB 5; T2- T1)	RA vs VD	t=2.04; p=0.04	t=3.66; p<0.01	n.s.	n.s.
	VD vs D	n.s.	t=2.80; p=0.01	n.s.	n.s.
Belief regarding the effectiveness of treatment (T2–T1)	RA vs VD	n.s.	n.s.	n.s.	t=-2.07; p=0.04
Belief about the attitude of other people to people with this disease (IRB 11; T2–T1)	RA vs VD	n.s.	n.s.	n.s.	t=-2.27; p=0.03
Belief regarding the comparison of disease severity with other people (IRB 13; T2-T1)	RA vs VD	n.s.	n.s.	n.s.	t=-2.05; p=0.04
Harm	RA vs VD	t=1.99; p=0.05	n.s.	n.s.	t=2.19; p=0.03
Importance	VD vs D	t=2.63; p=0.01	n.s.	n.s.	n.s.
Challenge (T2-T1)	RA vs VD	n.s.	n.s.	t=-2.18; p=0.03	n.s.
Harm (T2-T1)	RA vs VD	n.s.	n.s.	n.s.	t=-2.15; p=0.03
Threat (T2-T1)	RA vs VD	t=-2.69; p=0.01	n.s.	t=-3.61; p<0.01	t=-2.12; p=0.04
Age	RA vs D	t=1.95; p=0.05	n.s.	n.s.	n.s.
	VD vs D	t=2.29; p=0.02	n.s.	n.s.	t=3.05; p<0.01
	RA vs VD	n.s.	n.s.	n.s.	t=1.96; p=0.05
Number of hospitalizations	VD vs D	n.s.	t=1.97; p=0.05	n.s.	n.s.

*Note: only statistically significant interactions are presented in the table. RA – rheumatoid arthritis; VD – vascular diseases; D – diabetes; IRB – illness-related belief; n.s. – not signifficant
