# Supplementary material for: Age, Cognitive Factors, and Acceptance of Living with the Disease in Rheumatoid Arthritis: The Short-Term Perspective
Source: Int J Environ Res Public Health. 2022 Mar 7;19(5):3136. doi: 10.3390/ijerph19053136 (PMC8910175; doi:10.3390/ijerph19053136)
Supplement: Supplementary file 1 [file ijerph-19-03136-s001.zip › Supplementary File S2 IRAS.pdf]

Illness-Related Appraisals Scale

Revised Version

A disease may have different meanings for each person, therefore people think differently about their illnesses. The purpose of this questionnaire is to find out how important your illness is to you and how you feel about your illness.

Below are examples of the different ways of thinking about your disease. Read each one carefully and indicate how often you thought about your illness in this way over the last four weeks.

During the last four weeks, how often have you thought that….	I hardly thought so at all	I rarely thought that	Sometimes I thought so	I've often thought so	I thought so almost all the time
1. this disease has taken away something that was precious to you?					
2. this disease is an injustice that has happened to you?					
3. this disease has had some benefits for you?					
4. this disease is a difficulty in life that you have to deal with?					
5. thanks to this disease, you can sometimes obtain material benefit (e.g. a disability pension or material assistance)?					
6. this disease has helped you change for the better?					
7. it is not fair that this has happened to you?					
8. thanks to this disease, you can get something beneficial for yourself from other people?					
9. this disease is an adversity you have to face?					
10. thanks to this disease, you now appreciate each moment more?					
11. this disease sometimes benefits you?					
12. this disease is an opponent that you have to fight?					
13. this disease can still do you a lot more harm?					
14. this disease helped you better understand what is really important in life?					
15. this disease has irreversibly robbed you of something important?					
16. this disease will bring you many more dangers?					
17. this disease took away your previous way of life?					
18. this disease is a misfortune that fell on you for no reason?					
19. this disease is a test in life that you have to deal with?					
20. this illness can be a convenient excuse for you in certain situations (e.g., to avoid performing certain duties)?					
21. this disease helped you discover the true value of life?					
22. this disease could yet cost you much in life?					
23. this disease is a difficult life situation that you have to deal with?					
24. this disease can cause a lot of harm in the future?					
25. this disease made you value life more?					

During the last four weeks, *how often have you thought that….*	I hardly thought so at all	I rarely thought that	Sometimes I thought so	I've often thought so	I thought so almost all the time
26. this disease is a twist of fate that you did not deserve?					
27. through this disease you have lost the possibility of realizing plans you had for your life?					
28. your future is threatened by this disease?					
29. this disease means you can no longer live as before?					
30. this disease is a misfortune that happened to you?					

Please indicate to what extent you agree with the following statements.

Your illness:	I definitely agree	I tend to agree	Neither yes nor no	I rather disagree	I strongly disagree
31. disrupts your psychological balance					
32. disrupts your life balance					
33. is a small thing for you					
34. has little meaning to you					
35. painfully impedes your life					

© Copyright by Konrad Janowski, Stanisława Steuden, Daniel Pankowski
Warsaw, 2019
Use only with the consent of the authors
