# Supplementary material for: Age, Cognitive Factors, and Acceptance of Living with the Disease in Rheumatoid Arthritis: The Short-Term Perspective
Source: Int J Environ Res Public Health. 2022 Mar 7;19(5):3136. doi: 10.3390/ijerph19053136 (PMC8910175; doi:10.3390/ijerph19053136)
Supplement: Supplementary file 1 [file ijerph-19-03136-s001.zip › Supplementary File S9.pdf]

Supplementary File 9: T1-T2 and T1 correlation matrix

Supplementary File 9a1: T1 – T2 IRBs correlation matrix

	T2 IRB 1	T2 IRB 2	T2 IRB 3	T2 IRB 4	T2 IRB 5	T2 IRB 6	T2 IRB 7	T2 IRB 8	T2 IRB 9	T2 IRB 10	T2 IRB 11	T2 IRB 12	T2 IRB 13
T1 IRB 1	0.266**												
T1 IRB 2		0.413***											
T1 IRB 3			0.567***										
T1 IRB 4				0.588***									
T1 IRB 5					0.556***								
T1 IRB 6						0.547***							
T1 IRB 7							0.692***						
T1 IRB 8								0.389***					
T1 IRB 9									0.420***				
T1 IRB 10										0.316***			
T1 IRB 11											0.542***		
T1 IRB 12												0.695***	
T1 IRB 13													0.523***

IRB – Illness-related belief; *-p<0.05; **-p<0.01; ***-p<0.001

Supplementary File 9a2 T1 – T2 cognitive appraisals correlation matrix

	T2 Loss	T2 Harm	T2 Benefit	T2 Challenge	T2 Value	T2 Threat	T2 Importance
T1 Loss	0.734***						
T1 Harm		0.777***					
T1 Benefit			0.646***				
T1 Challenge				0.707***			
T1 Value					0.771***		
T1 Threat						0.713***	
T1 Importance							0.583***

*-p<0.05; **-p<0.01; ***-p<0.001

Supplementary File 9a2 T1 – T2 acceptance of life with the disease correlation matrix

	T2 Acceptance: Global score	T2 Satisfaction with Life Despite the Disease	T2 Reconcilement with the Disease	T1 Self-distancing from the Disease
T1 Acceptance: Global score	0.743***			
T1 Satisfaction with Life Despite the Disease		0.788***		
T1 Reconcilement with the Disease			0.692***	
T1 Self-distancing from the Disease				0.624***

*-p<0.05; **-p<0.01; ***-p<0.001

Supplementary File 9b T1 – T2 IRBs correlation matrix in RA patients

	T2 IRB 1	T2 IRB 2	T2 IRB 3	T2 IRB 4	T2 IRB 5	T2 IRB 6	T2 IRB 7	T2 IRB 8	T2 IRB 9	T2 IRB 10	T2 IRB 11	T2 IRB 12	T2 IRB 13
T1 IRB 1	0.320**												
T1 IRB 2		0.394***											
T1 IRB 3			0.546***										
T1 IRB 4				0.666***									
T1 IRB 5					0.551***								
T1 IRB 6						0.557***							
T1 IRB 7							0.755***						
T1 IRB 8								0.379**					
T1 IRB 9									0.417***				
T1 IRB 10										0.351**			
T1 IRB 11											0.615***		
T1 IRB 12												0.746***	
T1 IRB 13													0.501***

IRB – Illness-related belief; *-p<0.05; **-p<0.01; ***-p<0.001

Supplementary File 9b2 T1 – T2 cognitive appraisals correlation matrix in RA patients

	T2 Loss	T2 Harm	T2 Benefit	T2 Challenge	T2 Value	T2 Threat	T2 Importance
T1 Loss	0.715***						
T1 Harm		0.773***					
T1 Benefit			0.738***				
T1 Challenge				0.691***			
T1 Value					0.757***		
T1 Threat						0.713***	
T1 Importance							0.539***

*-p<0.05; **-p<0.01; ***-p<0.001

Supplementary File 9b2 T1 – T2 acceptance of life with the disease correlation matrix in RA patients

	T2 Acceptance: Global score	T2 Satisfaction with Life Despite the Disease	T2 Reconcilement with the Disease	T1 Self-distancing from the Disease
T1 Acceptance: Global score	0.778***			
T1 Satisfaction with Life Despite the Disease		0.824***		
T1 Reconcilement with the Disease			0.726***	
T1 Self-distancing from the Disease				0.685***

*-p<0.05; **-p<0.01; ***-p<0.001

Supplementary File 9c T1 – T2 IRBs correlation matrix in VD patients

	T2 IRB 1	T2 IRB 2	T2 IRB 3	T2 IRB 4	T2 IRB 5	T2 IRB 6	T2 IRB 7	T2 IRB 8	T2 IRB 9	T2 IRB 10	T2 IRB 11	T2 IRB 12	T2 IRB 13
T1 IRB 1	0.279												
T1 IRB 2		0.568**											
T1 IRB 3			0.683***										
T1 IRB 4				0.510**									
T1 IRB 5					0.656***								
T1 IRB 6						0.423*							
T1 IRB 7							0.526**						
T1 IRB 8								0.434*					
T1 IRB 9									0.234				
T1 IRB 10										0.226			
T1 IRB 11											0.176		
T1 IRB 12												0.337	
T1 IRB 13													0.525**

IRB – Illness-related belief; *-p<0.05; **-p<0.01; ***-p<0.001

Supplementary File 9c2 T1 – T2 cognitive appraisals correlation matrix in VD patients

	T2 Loss	T2 Harm	T2 Benefit	T2 Challenge	T2 Value	T2 Threat	T2 Importance
T1 Loss	0.726***						
T1 Harm		0.748***					
T1 Benefit			0.354				
T1 Challenge				0.659***			
T1 Value					0.748***		
T1 Threat						0.692***	
T1 Importance							0.639***

*-p<0.05; **-p<0.01; ***-p<0.001

Supplementary File 9c2 T1 – T2 acceptance of life with the disease correlation matrix in VD patients

	T2 Acceptance: Global score	T2 Satisfaction with Life Despite the Disease	T2 Reconcilement with the Disease	T1 Self-distancing from the Disease
T1 Acceptance: Global score	0.567**			
T1 Satisfaction with Life Despite the Disease		0.654***		
T1 Reconcilement with the Disease			0.475*	
T1 Self-distancing from the Disease				0.316

*-p<0.05; **-p<0.01; ***-p<0.001

Supplementary File 9d T1 – T2 IRBs correlation matrix in diabetes patients

	T2 IRB 1	T2 IRB 2	T2 IRB 3	T2 IRB 4	T2 IRB 5	T2 IRB 6	T2 IRB 7	T2 IRB 8	T2 IRB 9	T2 IRB 10	T2 IRB 11	T2 IRB 12	T2 IRB 13
T1 IRB 1	0.190												
T1 IRB 2		0.209											
T1 IRB 3			0.409*										
T1 IRB 4				0.410*									
T1 IRB 5					0.335								
T1 IRB 6						0.563**							
T1 IRB 7							0.682***						
T1 IRB 8								0.327					
T1 IRB 9									0.735***				
T1 IRB 10										0.255			
T1 IRB 11											0.702***		
T1 IRB 12												0.872***	
T1 IRB 13													0.469*

IRB – Illness-related belief; *-p<0.05; **-p<0.01; ***-p<0.001

Supplementary File 9d2 T1 – T2 cognitive appraisals correlation matrix in diabetes patients

	T2 Loss	T2 Harm	T2 Benefit	T2 Challenge	T2 Value	T2 Threat	T2 Importance
T1 Loss	0.752***						
T1 Harm		0.846***					
T1 Benefit			0.595**				
T1 Challenge				0.771***			
T1 Value					0.835***		
T1 Threat						0.705***	
T1 Importance							0.357

*-p<0.05; **-p<0.01; ***-p<0.001

Supplementary File 9d2 T1 – T2 acceptance of life with the disease correlation matrix in diabetes patients

	T2 Acceptance: Global score	T2 Satisfaction with Life Despite the Disease	T2 Reconcilement with the Disease	T1 Self-distancing from the Disease
T1 Acceptance: Global score	0.738***			
T1 Satisfaction with Life Despite the Disease		0.745***		
T1 Reconcilement with the Disease			0.780***	
T1 Self-distancing from the Disease				0.699***

*-p<0.05; **-p<0.01; ***-p<0.001

Supplementary File 9. T1 (*N*=83) correlation matrix

	IRB 1	IRB 2	IRB 3	IRB 4	IRB 5	IRB 6	IRB 7	IRB 8	IRB 9	IRB 10	IRB 11	IRB 12	IRB 13	Loss	Harm	Benefit	Challenge	Value	Threat	Importance	ALD: Satisfaction	ALD: Reconcilement	ALD: Distancing
IRB 2	0.462***																						
IRB 3	0.494***	0.633***																					
IRB 4	0.168	0.426***	0.314**																				
IRB 5	0.278**	0.46***	0.485***	0.392***																			
IRB 6	0.497***	0.323**	0.276*	0.247*	0.27*																		
IRB 7	-0.007	0.198	0.078	0.258*	0.169	-0.108																	
IRB 8	0.22*	0.028	-0.010	-0.023	0.031	0.032	-0.085																
IRB 9	-0.074	0.033	0.130	0.082	0.288**	-0.004	0.175	0.180															
IRB 10	0.065	0.229*	0.208	0.244*	0.065	0.019	0.161	0.143	0.242*														
IRB 11	0.051	0.125	0.174	0.211	0.093	-0.116	0.228*	0.183	0.237*	0.426***													
IRB 12	-0.23*	-0.009	-0.047	0.37***	-0.020	-0.159	0.160	-0.072	0.061	0.148	0.319**												
IRB 13	-0.054	0.352**	0.348**	0.364***	0.266*	-0.041	0.170	0.235*	0.246*	0.373***	0.407***	0.265*											
Loss	0.122	0.396***	0.288**	0.305**	0.233*	0.173	0.304**	0.111	0.219*	0.434***	0.255*	0.132	0.309**										
Harm	-0.052	0.175	0.114	0.187	0.226*	0.147	0.211	0.106	0.304**	0.347**	0.244*	0.34**	0.275*	0.691***									
Benefit	-0.248*	-0.124	-0.076	0.157	-0.014	-0.040	0.441***	-0.180	0.251*	-0.030	0.069	0.295**	0.008	0.175	0.27*								
Challenge	0.167	0.402***	0.266*	0.325**	0.371***	0.277*	0.223*	0.017	0.160	0.247*	0.182	0.238*	0.282**	0.622***	0.492***	0.135							
Value	-0.019	0.281*	0.085	0.184	0.140	0.098	0.349**	0.045	0.115	0.097	0.138	0.144	0.059	0.486***	0.393***	0.477***	0.594***						
Threat	0.23*	0.525***	0.41***	0.24*	0.292**	0.225*	0.179	0.091	0.194	0.403***	0.177	0.151	0.323**	0.837***	0.69***	0.103	0.61***	0.392***					
Importance	0.154	0.489***	0.372***	0.257*	0.363***	0.140	0.078	0.110	0.22*	0.384***	0.082	0.059	0.282**	0.61***	0.484***	0.064	0.473***	0.268*	0.648***				
ALD: Satisfaction	-0.111	0.018	0.062	0.027	0.029	-0.136	0.112	0.030	0.137	0.397***	0.127	0.226*	0.31**	0.472***	0.497***	0.060	0.172	-0.093	0.458***	0.409***			
ALD: Reconcilement	-0.236*	-0.131	-0.087	-0.033	0.004	-0.245*	0.084	0.076	0.224*	0.295**	0.28*	0.338**	0.298**	0.381***	0.535***	0.184	0.136	0.026	0.357**	0.22*	0.782***		

ALD: Distancing	-0.086	0.173	0.219*	0.133	0.170	-0.144	0.069	0.079	0.111	0.297**	0.177	0.264*	0.5***	0.47***	0.516***	0.005	0.28*	0.038	0.534***	0.486***	0.767***	0.673***	
ALD: Global score	-0.153	0.030	0.080	0.051	0.077	-0.188	0.097	0.067	0.169	0.364***	0.209	0.299**	0.409***	0.488***	0.565***	0.085	0.218*	-0.013	0.499***	0.417***	0.938***	0.889***	0.901***
