# Supplementary figures and images for: Age, Cognitive Factors, and Acceptance of Living with the Disease in Rheumatoid Arthritis: The Short-Term Perspective
Source: Int J Environ Res Public Health. 2022 Mar 7;19(5):3136. doi: 10.3390/ijerph19053136 (PMC8910175; doi:10.3390/ijerph19053136)

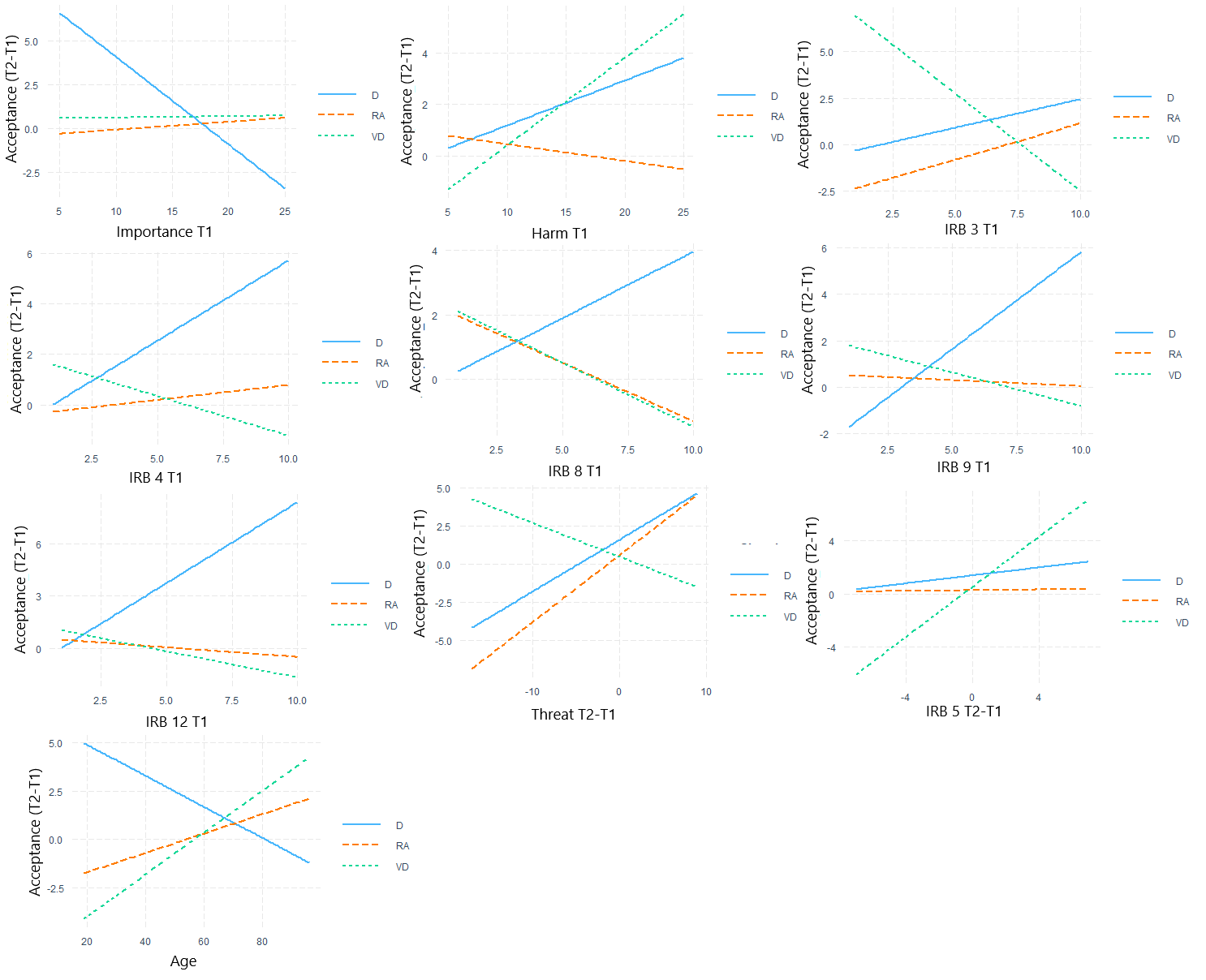

Supplement: Supplementary file 1 [file ijerph-19-03136-s001.zip › Supplementary File S12.tiff]

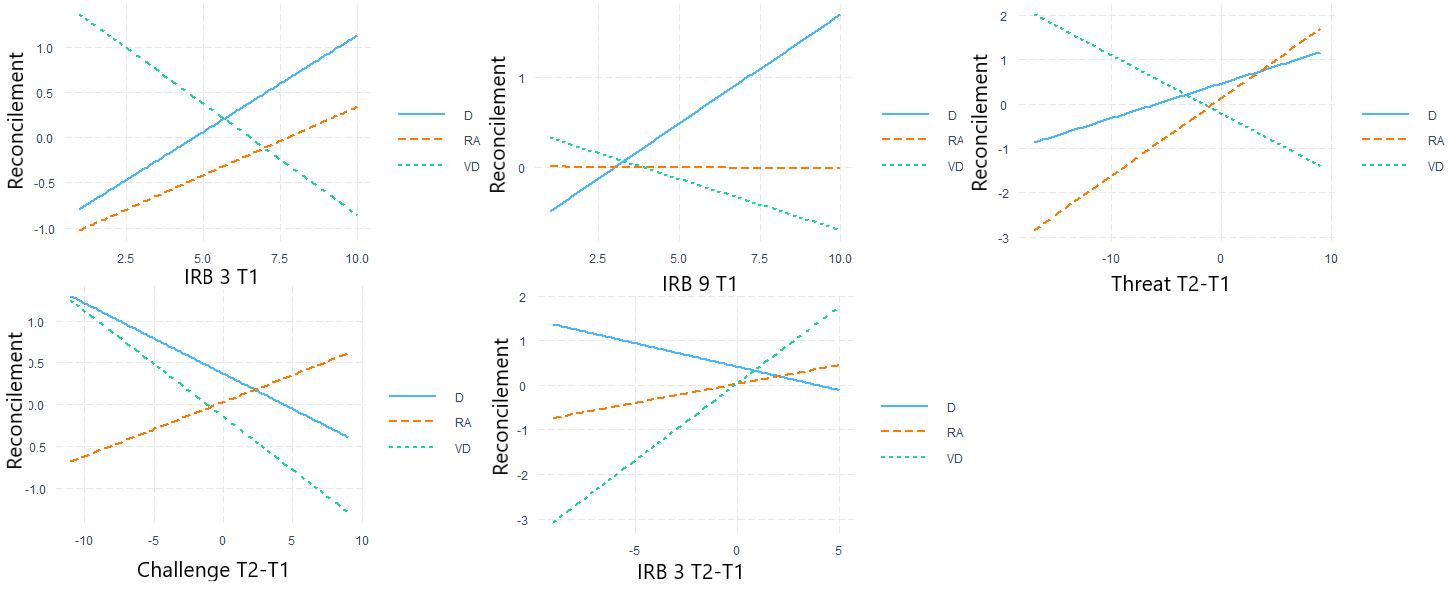

Supplement: Supplementary file 1 [file ijerph-19-03136-s001.zip › Supplementary File S13.tiff]

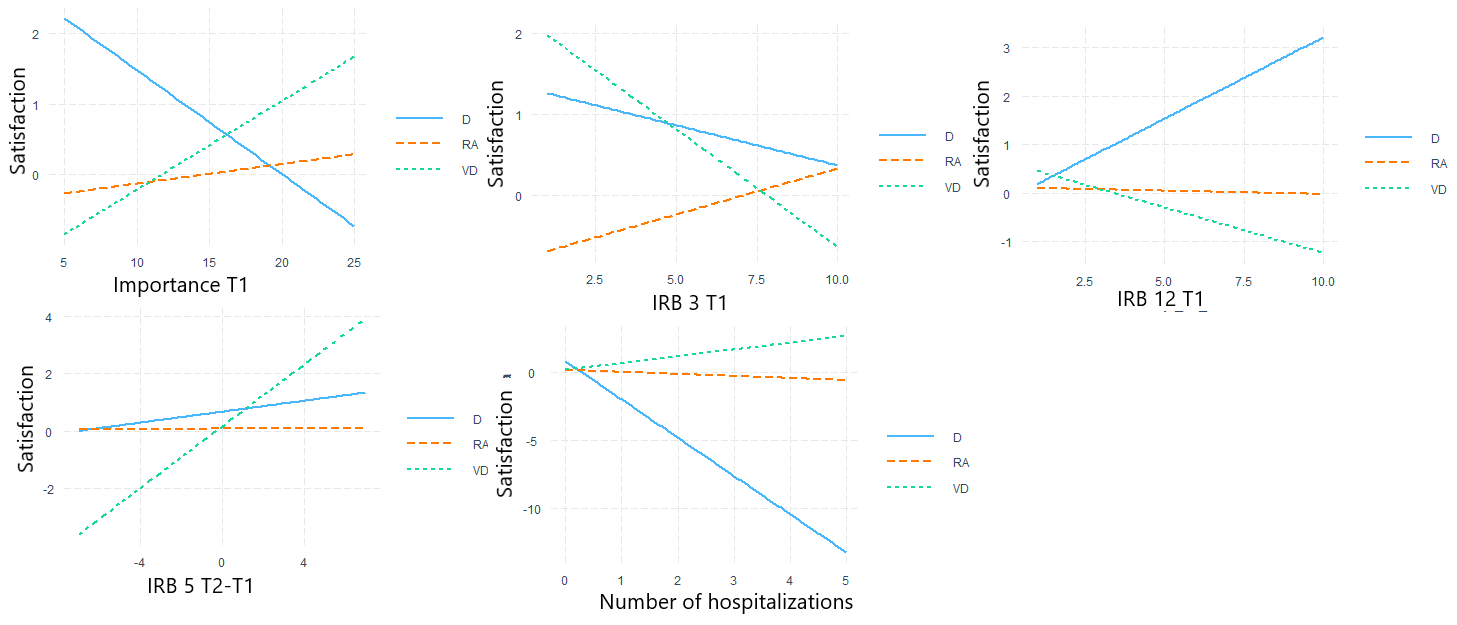

Supplement: Supplementary file 1 [file ijerph-19-03136-s001.zip › Supplementary File S14.tiff]

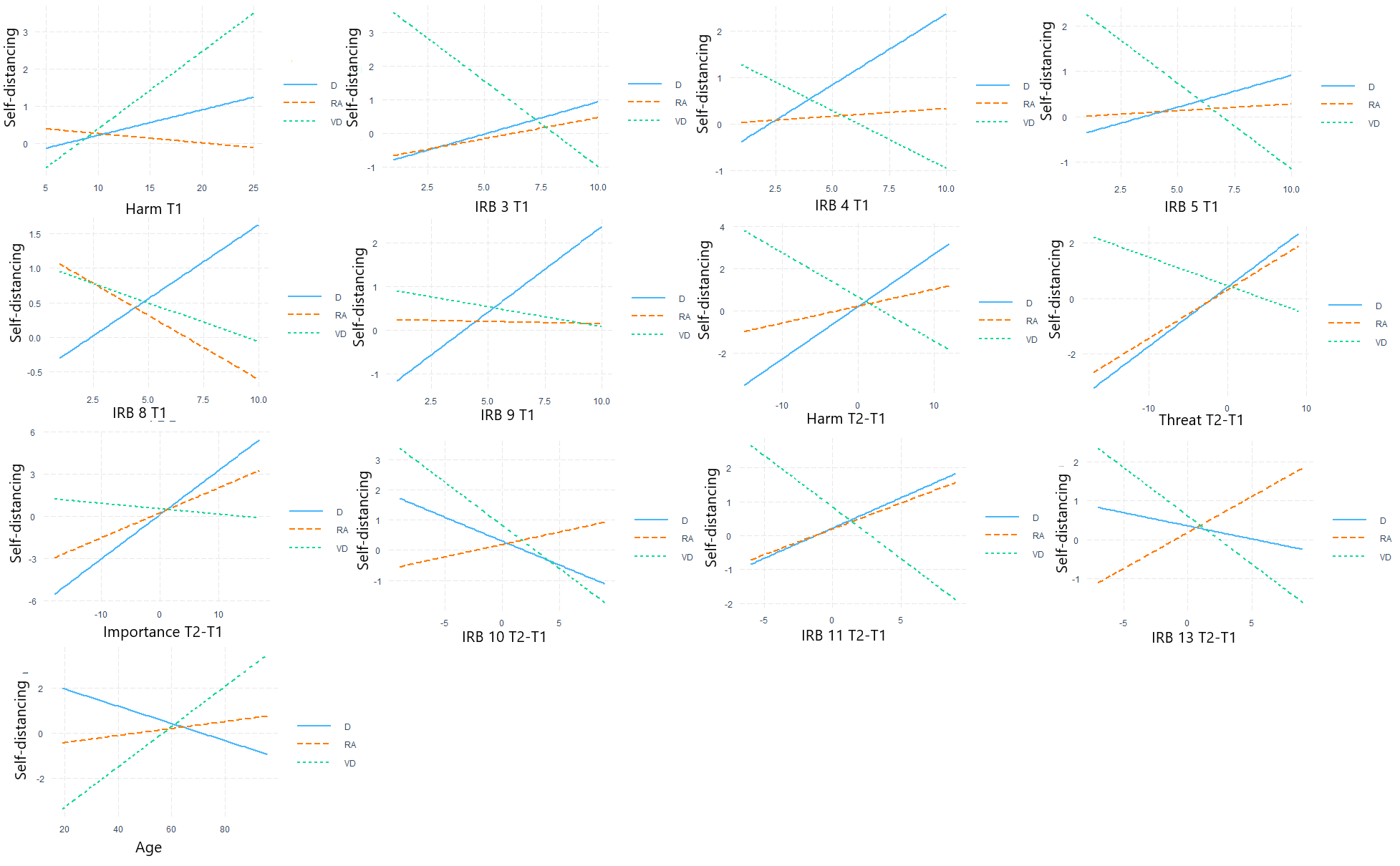

Supplement: Supplementary file 1 [file ijerph-19-03136-s001.zip › Supplementary File S15.tiff]

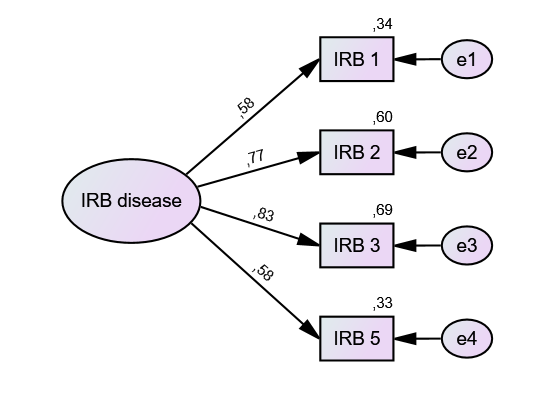

Supplement: Supplementary file 1 [file ijerph-19-03136-s001.zip › Supplementary File S3 IRB disease.tiff]

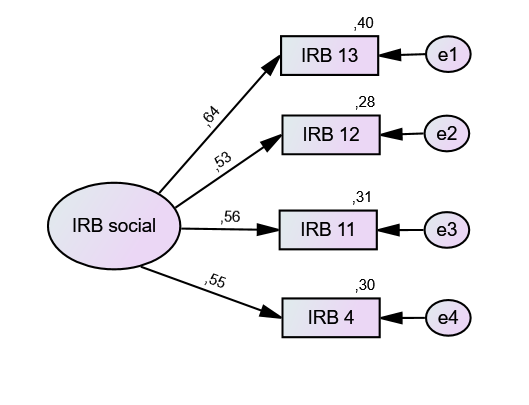

Supplement: Supplementary file 1 [file ijerph-19-03136-s001.zip › Supplementary File S4 IRB social.tiff]

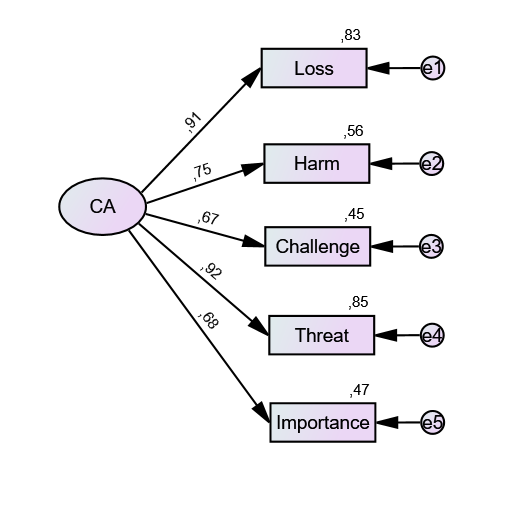

Supplement: Supplementary file 1 [file ijerph-19-03136-s001.zip › Supplementary File S5 CA.tiff]

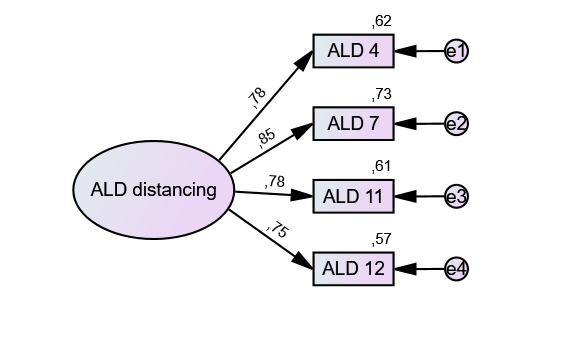

Supplement: Supplementary file 1 [file ijerph-19-03136-s001.zip › Supplementary File S6 ALD distancing.tiff]

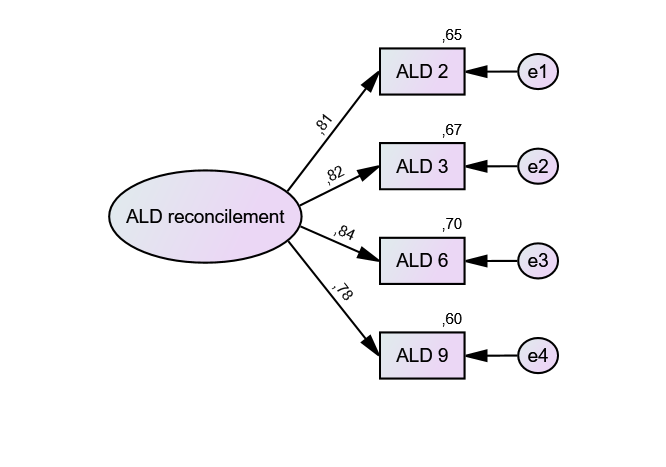

Supplement: Supplementary file 1 [file ijerph-19-03136-s001.zip › Supplementary File S7 ALD reconcilement.tiff]

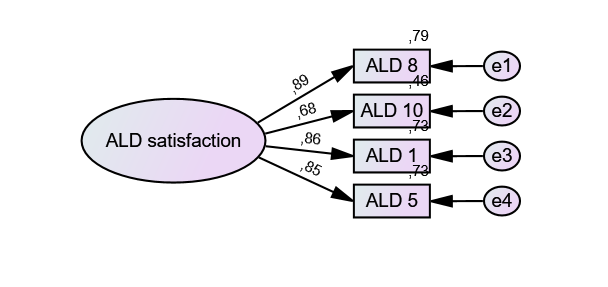

Supplement: Supplementary file 1 [file ijerph-19-03136-s001.zip › Supplementary File S8 ald satsfaction.tiff]
